# Factors Predictive of Protracted Course of Radiation Therapy in Patients Treated with Definitive Chemoradiation for Cervical Cancer

**DOI:** 10.7759/cureus.558

**Published:** 2016-04-04

**Authors:** Mark Zaki, Michael Dominello, Robert Morris, Steven Miller

**Affiliations:** 1 Radiation Oncology, Detroit Medical Center; 2 Department of Oncology, Division of Radiation Oncology, Karmanos Cancer Center; 3 Department of Oncology, Division of Radiation Oncology, Wayne State University School of Medicine; 4 Gynecologic Oncology, Karmanos Cancer Center, Wayne State University School of Medicine; 5 Department of Oncology, Wayne State University School of Medicine

**Keywords:** cervical cancer

## Abstract

**Background:**

There is a benefit to completing definitive chemoradiotherapy (CRT) for cervical cancer within 56 days. However, many patients experience delays due to missed radiation treatments that prolong the overall course of therapy. In order to improve patient care, we performed a quality improvement project to determine factors predictive of protracted treatment and develop strategies to enable timely treatment completion.

**Methods:**

Seventy-one patients treated for cervical cancer with CRT were identified. Medical records were reviewed to gather demographic, clinical, and treatment data. Prolonged treatment was defined as >56 days per the American Brachytherapy Society guidelines. The following variables were evaluated using paired t-tests and univariate logistic regression: demographics, Intensity Modulated Radiotherapy (IMRT) versus conventional radiation technique, use of a boost, time to stent placement, time to first brachytherapy (BT), and genitourinary (GU) or gastrointestinal (GI) toxicity.

**Results:**

The median treatment length for all patients was 59 days. Factors associated with prolonged treatment were time to cervical stent placement (p=0.001), delay ≥2 days between final external beam radiation therapy (EBRT) and initial BT (p=0.0195), any grade GU toxicity (p=0.0007), or GI toxicity (p=0.0002), and the presence of a boost (p=0.0006). Age, stage, and IMRT versus conventional technique were not associated with protracted treatment.

**Conclusion:**

In this series of patients, acute toxicity, increased time to cervical stent placement, and time to first BT treatment were associated with prolonged treatment time. The patients who completed treatment in ≤56 days had a lower average time to cervical stent placement, 27 versus 31 days. Our results suggest that cervical stent placement during week four of treatment can enhance patient care and improve outcomes.

## Introduction

Previous studies have demonstrated a benefit to completing definitive chemoradiotherapy (CRT) for cervical cancer within an eight-week period [1-8]. Furthermore, the American Brachytherapy Society (ABS) task group recommends finishing CRT within 56 days from the start of treatment [9]. Current Radiation Therapy Oncology Group (RTOG) and Gynecologic Oncology Group (GOG) cervical cancer protocols utilizing CRT recommend that treatment be completed within 56 days (eight weeks) [10-11] . Despite the well-established detriment of protracted treatment course, patients may, for various reasons, still experience delays that prolong the course of their treatment.

To determine potential causes of prolonged treatment, we investigated many possible factors. An important variable we examined was the time to cervical stent insertion. To facilitate insertion of a tandem, a cervical stent is typically inserted on the first day of, or prior to the start of, high-dose-rate brachytherapy (BT) treatment [12]. At many institutions including our own, a cervical stent is placed one or more days prior to the first BT treatment, though optimal time of placement is unknown. Placement early on, as the cervix is responding to therapy, may compromise the positioning of the stent, suture integrity, and dosimetry at the time of BT planning, whereas late placement may prolong overall treatment length. Our objective was to identify factors that may predict for prolonged treatment time, including time of stent placement, in order to implement strategies to enhance the quality of patient care.

## Materials and methods

After institutional review board (IRB) approval, seventy-one consecutive patients treated from 2008 to 2013 for cervical cancer with CRT followed by BT at our institution were identified. Medical records were reviewed to gather demographic, clinical, and treatment data. Informed consent was obtained from all participants. Prolonged treatment was defined as >56 days as per the American Brachytherapy Society (ABS) guidelines [13].

Outcomes were not analyzed in this report as our outcomes are expected to represent existing data. Therefore, we limited the scope of this project to analyze the demographic, clinical, and treatment-related factors associated with treatment prolongation in order to recommend quality improvement measures.

Total treatment time was calculated as the number of days from the date of the first radiation treatment to the last radiation treatment, including both external beam radiation therapy (EBRT) and BT. Treatment breaks were defined as any single break in radiation treatment ≥3 days (excluding weekends or holidays) or multiple breaks during the course of radiation resulting in ≥5 missed treatments. Toxicity was evaluated by assessments performed on weekly on-treatment visits. 

Data was analyzed using SAS V. 9.2 (SAS Inc., Cary, NC). Treatment time was transformed into a dichotomous variable (≤56 and >56 days). The variables related to increased treatment time were evaluated using paired t-tests and univariate logistic regression. Important univariate variables were then fit into a multivariable logistic model.

## Results

In our series of patients, the median age was 50. Thirty-nine percent of patients had FIGO stage I disease, 39% had stage II, 16% had stage III, and 6% had stage IV. (FIGO is International Federation of Gynecology and Obstetrics). Median EBRT dose was 45 Gy in 25 fractions of 1.8 Gy to the pelvis ± para-aortic lymph nodes, with a 5.4 Gy sidewall or parametrial boost. The median BT dose was 27.5 Gy in 5 fractions. Sixty-six percent were treated with 3-D conformal radiation and 34% using IMRT. Sixty-eight women had high-dose-rate BT with a ring and tandem applicator, whereas three underwent an interstitial implant. Table 1 highlights the characteristics of patients in our cohort. 

**Table 1:** Patient characteristics EBRT- external beam radiation therapy. HDR - high-dose rate brachytherapy. IMRT - intensity modulated radiation therapy.

Patient Characteristics	
Median age	50
Stage	
I	28 (39%)
II	28 (39%)
III	11 (16%)
IV	4 (6%)
EBRT dose (median)	45 Gy
Boost dose (median)	5.4 Gy
HDR dose (median)	27.5 Gy
4-field technique	47 (66%)
IMRT	24 (34%)
Ring and tandem	68 (96%)
Interstitial implant	3 (4%)
Length of treatment (median)	59 days
First treatment to cervical stent placement (median)	29 days
First EBRT to HDR (median)	38 days

For the 68 patients requiring a cervical stent prior to application of the ring and tandem, the median time from start of EBRT to the cervical stent placement and first BT was 29 and 38 days, respectively. The median treatment length for all patients was 59 days. Fifty-one percent of patients had one or more breaks in treatment and 59% of those breaks were either due to social issues or non-compliance.

**Figure 1:**
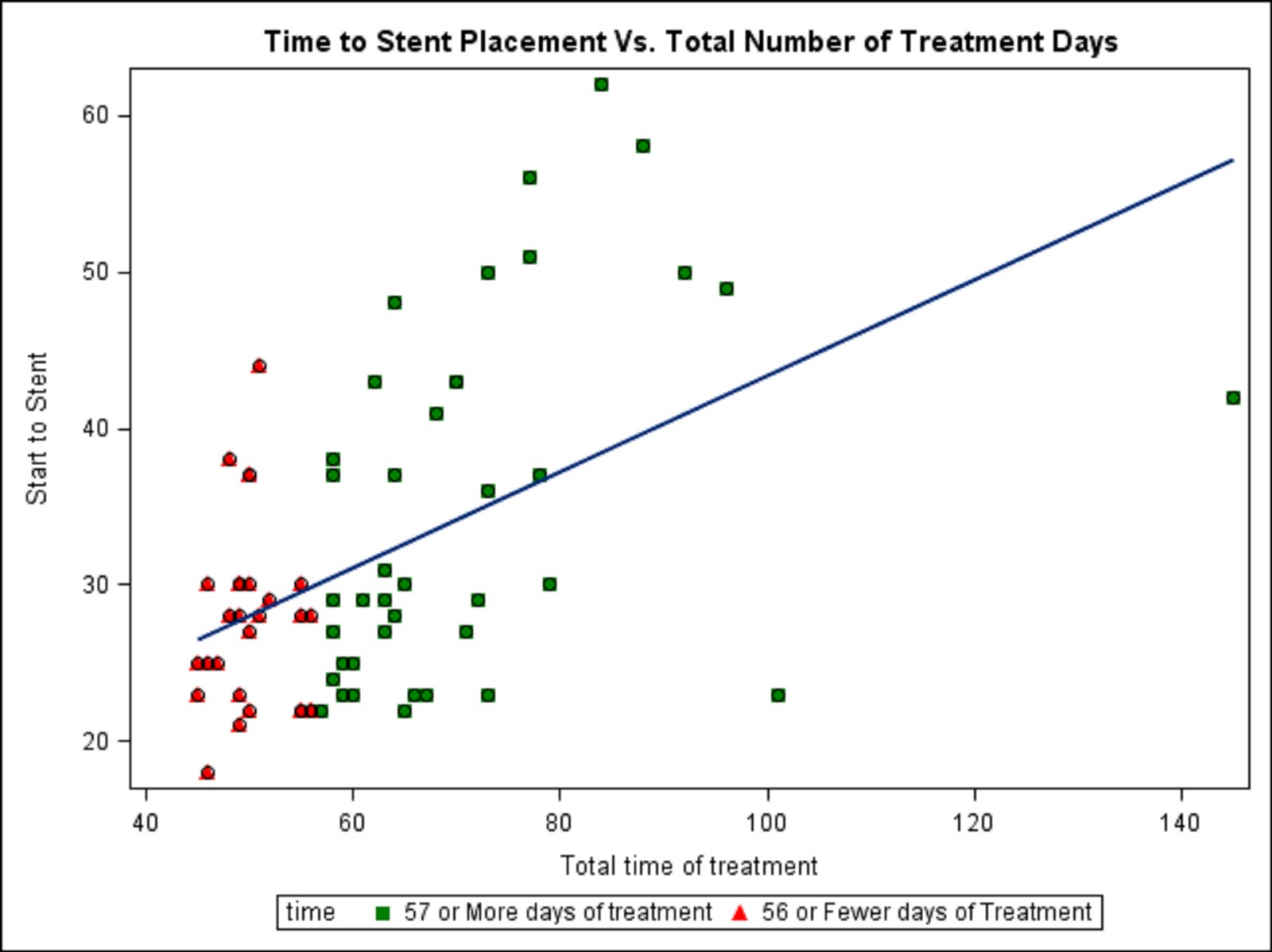
Graphical representation of time from first external beam radiation treatment to cervical stent placement versus total time of treatment, including a regression plot.

In addition to the time of stent placement, the following variables were evaluated: age, race, distance to cancer center, use of IMRT versus conventional radiation technique, use of a boost, time to first BT, and GU or GI toxicity. Factors associated with prolonged treatment time were time to cervical stent placement (p=0.001), delay ≥2 days between last fraction of EBRT and delivery of first BT (p=0.0195), any grade GU toxicity (p=0.0007), or GI toxicity (p=0.0002), and the presence of a boost (p=0.0006). Age, race, stage, distance to cancer center, and radiation technique (IMRT v. 3-D) were not significantly associated with prolonged treatment time. Figure 1 shows the relationship between the timing of cervical stent placement and overall treatment time.

When excluding patients with any radiation treatment breaks, the following factors remained associated with prolonged treatment: time to cervical stent placement, with a mean of 31 days versus 27 days (p=0.05), and time to first BT treatment, with a mean of 47 days versus 34 days in patients completing treatment in >56 and ≤56 days respectively (p=0.01). 

## Discussion

Overall treatment length of >56 days is detrimental for patients receiving definitive CRT therapy for cervical cancer [1-8], yet many patients do not complete treatment in this acceptable time period. Prolonged treatment duration has been shown to decrease pelvic tumor control by an estimated 0.3-1.6% per day of prolongation [1, 2, 4, 7-8]. Tumor regrowth by repopulation of surviving tumor cells during fractionated radiotherapy is the reason why local control rate decreases with time [14]. This radiobiological mechanism is critical in cervical cancer which demonstrates accelerated repopulation with a short onset time [15], magnifying the importance of reducing treatment time in this disease.

Treatment breaks, whatever their cause, are a significant source of protracted treatment time. Every effort should be made to limit preventable breaks in therapy. This involves counseling patients on the importance of maintaining compliance to the established treatment schedule. Selected patients may also benefit from a social work evaluation to optimize external factors and social issues, as well as a psychiatric evaluation for management of co-morbid psychiatric illnesses such as depression or anxiety disorders [16-17]. This evidently plays a central role in compliance to treatment. In our study cohort, 51% of patients experienced one or more treatment breaks and 59% of breaks were due to social issues or non-adherence.

Additionally, treatment breaks, whether physician-approved or not, can also be caused by treatment-related toxicity [18-19]. Patients experience variable levels of toxicity during CRT. In our cohort, any grade GU or GI toxicity was strongly correlated with prolonged treatment length (p=0.0007 and p=0.0002, respectively). It is crucial to promptly recognize and appropriately manage these side effects during the course of treatment to enable a patient to complete treatment in an acceptable time frame. Most common side effects include urinary and gastrointestinal irritation. Noninfectious dysuria can be managed with local analgesics such as phenazopyridine; nausea and vomiting is managed effectively with antiemetics such as odansetron or prochlorperazine; diarrhea is generally well-controlled with antidiarrheal medications such as loperamide or diphenoxylate/atropine [20].

The timing of BT must be carefully planned, using clinical and radiographic assessments. Early BT may not allow enough time for initial EBRT and chemotherapy to reduce the tumor size enough, compromising dosimetry at the time of BT; whereas BT started late in the course of BT may prolong the overall treatment time which may be detrimental to clinical outcomes. It has been shown that in addition to clinical examination, magnetic resonance imaging (MRI) is a valuable diagnostic tool that can often detect a significant amount of tumor reduction prior to the initial BT treatment and at each subsequent BT application [21-22]. The ABS task group recommends that highdose rate (HDR) BT commence after 39.6 Gy or 45 Gy with up to two BT treatments given per week during the conclusion of EBRT and that BT begins no earlier than approximately 20 Gy [9]. It should also be noted that the European study on MRI-guided brachytherapy in locally advanced cervical cancer (EMBRACE Study), endorsed by the Groupe Européen de Curiethérapie (GEC) and the European SocieTy for Radiotherapy & Oncology (ESTRO) (GEC-ESTRO) requires that all treatment be completed within 50 days [23]. Consistent with the ABS and GEC-ESTRO recommendations [9, 24], our study shows that cervical stent placement by the fourth week of EBRT would largely permit HDR BT to begin around 39.6 Gy, and is associated with satisfactory treatment duration.

A conceivable drawback of brachytherapy is that it is invasive, resource-intensive, and involves sedation or anesthesia. The recovery time from a brachytherapy procedure could also potentially contribute to a delay in overall treatment time. Completing all radiation treatments via EBRT is a promising, non-invasive way to limit treatment breaks, particularly in patients with poor performance status who are not suitable candidates for BT. Some investigators have published early experience with stereotactic body radiation therapy (SBRT) as a BT substitute in poor BT candidates. In retrospective series, SBRT provided adequate target coverage and satisfactory doses to organs at risk, with good toxicity profiles and clinical results on early follow-up [25-27]. Yet prospective data regarding SBRT boost are lacking and therefore it is not recommended as first-line treatment for patients who are BT candidates, outside of a clinical trial [28].

It is intuitive that reducing treatment breaks is fundamental, yet our quality improvement study importantly identifies time to cervical stent placement as an additional significant factor for achieving a satisfactory overall treatment time. When assessing patients in our cohort who completed treatment without a break in radiation, time to cervical stent placement was still a significant factor that predicted for completing treatment in ≤56 days. Scheduling cervical stent insertion during the fourth week of treatment is easily applicable and has a high likelihood of success at many institutions. We plan on implementing this intervention at our own institution. This key measure has the potential to significantly improve outcomes.

## Conclusions

In this series of patients, acute toxicity, increased time to cervical stent placement, and time to first BT treatment were associated with prolonged treatment time. Preventing unnecessary treatment breaks and treating acute toxicity effectively remains essential. Additionally, our results suggest that cervical stent placement during the fourth week of treatment may be effective in enhancing patient care and improving outcomes.
